# Strategies to Enhance Mesenchymal Stem Cell-Based Therapies for Acute Respiratory Distress Syndrome

**DOI:** 10.1155/2019/5432134

**Published:** 2019-11-22

**Authors:** Jibin Han, Yanmin Li, Yuanyuan Li

**Affiliations:** ^1^Department of Critical Care Medicine, First Hospital of Shanxi Medical University, No. 85, Jiefangnan Road, Taiyuan, 030001 Shanxi, China; ^2^Department of Anesthesiology, First Hospital of Shanxi Medical University, No. 85, Jiefangnan Road, Taiyuan, 030001 Shanxi, China

## Abstract

Acute respiratory distress syndrome (ARDS) is a multifaced disease characterized by the acute onset of hypoxemia, worsened pulmonary compliance, and noncardiogenic pulmonary edema. Despite over five decades of research, specific treatments for established ARDS are still lacking. MSC-based therapies have the advantage of targeting nearly all pathophysiological components of ARDS by means of a variety of secreted trophic factors, exerting anti-inflammatory, antioxidative, immunomodulatory, antiapoptotic, and proangiogenic effects, resulting in significant structural and functional recovery following ARDS in various preclinical models. However, the therapeutic efficacy of transplanted MSCs is limited by their poor engraftment and low survival rate in the injured tissues, major barriers to clinical translation. Accordingly, several strategies have been explored to improve MSC retention in the lung and enhance the innate properties of MSCs in preclinical models of ARDS. To provide a comprehensive and updated view, we summarize a large body of experimental evidence for a variety of strategies directed towards strengthening the therapeutic potential of MSCs in ARDS.

## 1. Introduction

ARDS is a catastrophic disease characterized by acute onset of hypoxic respiratory failure, noncardiogenic pulmonary edema, and decreased pulmonary compliance, which can subsequently trigger a cascade of serious complications and even progress to multiple organ failure. ARDS can result from various causes, including sepsis, multiple trauma, massive blood transfusion, pneumonia, aspiration, pulmonary contusion, and cardiopulmonary bypass. Abundant protein-rich fluid accumulated in the alveolar space due to diffuse alveolar-capillary barrier damage is the most prominent pathophysiological feature of patients with ARDS. Although comprehensive research has enabled clinicians to gain deep insight into the complex pathogenesis of ARDS, its incidence is still increasing [1]. The period prevalence of ARDS is 10.4% for patients admitted to ICUs, and the hospital mortality of patients with mild, moderate, and severe ARDS is 34.9%, 40.3%, and 46.1%, respectively [2]. Even though more patients are surviving ARDS due to advances in intensive care, these survivors of ARDS commonly suffer new or worsening brain dysfunction, cognitive impairment, anxiety symptoms, and physical limitations as well as increased readmission risk after hospital discharge in the following years, imposing substantial costs on the public health system [3].

Despite fifty years of research, there is still no specific therapy for ARDS. To date, therapeutic options remain confined to supportive care, including protective mechanical ventilation, prone-positioning ventilation, and fluid-conservative strategy. It is well established that mechanical ventilation with a lower tidal volume shortened the duration of mechanical ventilation and significantly decreased 28-day mortality [4]. Furthermore, early application of prolonged prone-positioning sessions significantly decreased 28-day and 90-day mortality for patients with severe ARDS [5]. However, mechanical ventilation carries a high risk for developing ventilation-induced lung injury (VILI) due to epithelial strain and stress from inhomogeneously injured lungs, and in turn, VILI exacerbates lung injury and stimulates an inflammatory reaction [6, 7]. Venovenous extracorporeal membrane oxygenation (VV-ECMO) is potentially a life-saving intervention to rescue patients with ARDS while avoiding overstretching the injured lungs [8], but nevertheless, the routine application of ECMO as a salvage therapy in patients with severe ARDS is still controversial [9]. Although early short-term use of a neuromuscular blockade in moderate to severe ARDS improved survival rates by decreasing markers of epithelial and endothelial injury and systemic inflammation [10], a variety of pharmacological therapies, including statins, aspirin, antioxidants, inhaled corticosteroids, beta-2 agonists, surfactants, and other anti-inflammatory drugs, have failed to show benefit [11]. To circumvent potential life-threatening complications and minimize the risk of mortality following ARDS, alternative therapeutic measures are urgently required to ameliorate lung injury and promote lung repair.

Theoretically, cell-based therapy can target multiple aspects of the pathophysiology underlying ARDS and may become a new kind of clinical therapy. Over recent years, cell therapy has been introduced in preclinical ARDS studies. A variety of cell types have been examined as promising candidates for potential therapeutic use, including embryonic stem cells (ESCs), induced pluripotent stem cells (iPSCs), mesenchymal stem cells (MSCs), pulmonary epithelial progenitor cells (EpPCs), and endothelial progenitor cells (EnPCs) [12]. Among these cell types, MSCs, also referred to as mesenchymal stromal cells, are of considerable interest as a potential candidate for the treatment of ARDS [13].

## 2. Mechanisms of MSCs in Treating ARDS

MSCs were initially isolated from the bone marrow by Friedenstein and his colleagues in the 1970s [14]. The minimum criteria for defining human MSCs proposed by the International Society for Cellular Therapy (ISCT) must meet the following requirements: MSCs are a plastic adherent cell; express cell surface marker of CD105, CD73, and CD90 and absence of CD45, CD34, CD14, and HLA-DR; and also, with the capacity to differentiate to osteoblasts, chondrocytes, and adipocytes under the appropriate condition [15, 16]. Apart from the bone marrow, MSCs can be harvested from a variety of sources, including adipose tissues and umbilical cord blood [17, 18]. A lot of studies show that the level of injected MSCs that differentiate into tissue-appropriate phenotypes is very low [19–21]. Increasing evidence support the notion that MSCs promote tissue recovery and regeneration via secreting a variety of paracrine factors, conferring anti-inflammatory, immunomodulatory, angiogenic, antifibrotic, antimicrobial, and structural reparative properties [22, 23]. Notably, MSCs possess an immunomodulatory property via inhibiting T cell proliferation and regulating B cell functions, as well as the ability to suppress the maturation of dendritic cells [24]. Furthermore, MSCs were shown to reprogram macrophages by secreting prostaglandin E2 to increase interleukin-10 production in a septic lung model [25]. Of additional interest, Islam et al. revealed that the mechanism behind the protective effect of MSCs against lung injury involved mitochondrial transfer to pulmonary alveoli [26].

## 3. Obstacles to Clinical Translation

**Figure 1:**
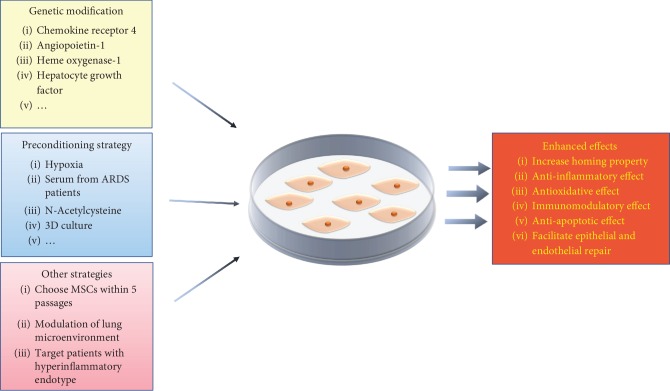
Schematic representation of the main strategies to enhance MSC therapeutic potential for ARDS.

Given that MSC-based therapy offers great promise in preclinical ARDS models, several phase I/II clinical trials have been conducted to assess the safety of human MSC injections in patients with ARDS. The study performed by Zheng et al. showed that no serious adverse events related to MSC administration were observed in twelve patients with ARDS [27]. Moreover, Wilson et al. also demonstrated that intravenous administration of MSCs was well tolerated in nine patients with moderate to severe ARDS [28]. Simonson et al. administered MSCs to two patients with severe ARDS and found that both patients demonstrated improved lung function and resolution of multiorgan failure [29]. Recently, the START study demonstrated that no hemodynamic or respiratory adverse events related to MSC infusion were observed over a 60-day follow-up period and the 28-day mortality was numerically higher in the MSC group than in the placebo but did not differ significantly between groups [30]. It is worth noting that the tendency toward harm in the START study may be attributed to the imbalances in the severity of illness and the wide variability in MSC viability. The completed clinical studies regarding the treatment of ARDS with MSCs were summarized in Table 1. Thus, there is an urgent need to advance the modest efficacy of MSCs observed in clinical trials. In addition, low mobilization of MSCs to the sites of injury and poor survival of transplanted MSCs in the harsh microenvironment are obstacles faced by clinical translation. Accordingly, several strategies have been explored to improve MSC retention in the lung and enhance the innate properties of MSCs in the preclinical model of ARDS (Figure 1).

## 4. Genetic Modification to Enhance MSC Potency

The strategy using genetic modification focusing on transfecting MSCs with beneficial genes has attracted the attention of researchers for its multiple advantages, not only enhancing the intrinsic ability of MSCs to migrate to the site of damage and facilitate tissue repair but also improving the capability of MSCs to survive in the harsh microenvironments. The candidate genes tested in preclinical ARDS models are summarized in Table 2.

## 5. Chemokine Receptor 4

Stromal-derived factor-1 (SDF-1) with its unique chemokine receptor 4 (CXCR4) has been found to play a vital role in directing the migration of MSCs. However, only a small portion of MSCs express CXCR4, and CXCR4 expression on the surface of MSCs significantly declined in the process of multiple ex vivo expansions. Low expression of CXCR4 in MSCs may account for their low efficiency in homing to damaged tissues, which limits the therapeutic effect. Yang et al. introduced CXCR4 into bone marrow MSCs via a lentiviral vector and showed that CXCR4 overexpression dramatically improved the chemotactic properties of MSCs in vitro [31]. Using a rat model of ALI induced by lipopolysaccharide (LPS), they also found that CXCR4 facilitated MSC homing to and colonization of injured lung tissues, and furthermore, CXCR4-MSCs offered an additional protective effect of attenuating LPS-induced ALI in rats [31].

## 6. E-Prostanoid 2 Receptor

Prostaglandin E2 (PGE2) is a vital inflammatory cytokine, and its biosynthesis is significantly increased in the inflammatory microenvironment. In particular, PGE2 promotes the migration of MSCs through activation of the E-prostanoid 2 (EP2) receptor in vitro [32]. Accordingly, it is apparently reasonable that PGE2 could be deemed a chemokine to facilitate the migration of MSCs by activating the EP2 receptor. Han et al. observed that PGE2 production markedly increased in the damaged lung tissues following LPS challenge [33]. In addition, they transduced the bone marrow-derived MSC-based EP2 gene by lentiviral vectors and tested their effects in a mouse model of LPS-induced ALI. Their results demonstrated that MSCs transfected with EP2 significantly facilitated the mobilization of MSCs to sites of lung injury and, furthermore, ameliorated both pulmonary inflammation and permeability [33].

## 7. Heme Oxygenase-1

Heme oxygenase-1 (HO-1) is a stress-response protein with antiapoptotic, anti-inflammatory, and antioxidative properties, protecting cells from injury and restoring homeostasis in various pathologic states. When using a Transwell system to coculture pulmonary microvascular endothelial cells (PVECs) injured by LPS and MSCs transfected with the HO-1 gene, the authors reported that HO-1-MSCs exerted an enhanced capacity to ameliorate LPS-induced inflammatory and oxidative damage in PVECs [34]. Chen et al. further tested the curative effects of bone marrow-derived MSCs overexpressing HO-1 via lentiviral vectors in the LPS-induced ALI rat model [35]. They demonstrated that HO-1-MSCs ameliorated cytokine levels in serum, the neutrophil counts and total protein concentration in bronchoalveolar lavage fluid, and the histological changes in the lung to a greater extent than unmodified MSCs [35].

## 8. Angiotensin-Converting Enzyme 2

Mounting evidence has suggested that angiotensin-converting enzyme 2 (ACE2) plays a prominent role in the physiological and pathological processes of the respiratory systems [36]. Intriguingly, ACE2 was reported to protect against lung injury by degrading the profibrotic peptide angiotensin (Ang) II [37]. Bone marrow-derived MSCs overexpressing the ACE2 gene via lentiviral vectors were demonstrated to have an enhanced ability to alleviate endothelial injury resulting from LPS stimulation [38]. He et al. demonstrated that the administration of ACE2-MSCs offered additional anti-inflammatory and endothelial-protective effects against LPS-induced lung injury in mice [39]. Furthermore, the study performed by Min et al. showed that MSCs and ACE2 had a synergistic therapeutic effect on bleomycin-induced ALI [40].

## 9. Hepatocyte Growth Factor

Hepatocyte growth factor (HGF), which was initially thought to be a potent mitogen for hepatocytes, is a candidate for boosting angiogenesis, engraftment, anti-inflammation, and antifibrosis due to its pleiotropic effect. Using a BALB/c mouse model of bronchiolitis obliterans, Cao et al. demonstrated that HGF-modified MSCs improved histopathological and biochemical markers in the recovery of allograft trachea histopathology [41]. Notably, Wang et al. suggested that HGF-modified bone marrow MSCs offered incremental benefits in radiation-induced ALI mice [42]. When compared with MSCs alone, HGF-MSCs significantly decreased the secretion and expression of proinflammatory cytokines as well as reduced the levels of profibrosis factors, while they markedly increased the expression of the anti-inflammatory cytokine interleukin-10 (IL-10) [42].

## 10. Angiopoietin-1

Angiopoietin-1 (Ang1), an angiogenic growth factor, acts as a crucial regulator of angiogenesis, vascular stabilization, and anti-inflammatory actions by improving vascular permeability and inhibiting leukocyte-endothelium interactions [43]. In a mouse model, the combination of MSC and Ang1 gene therapy exhibited a synergistic effect on alleviating LPS-induced lung injury, as reflected by improvement in the histopathological and biochemical indices [44]. In line with previous research, treatment with Ang1-MSCs resulted in a further improvement in both the recruitment of inflammatory cells into the lung and pulmonary vascular endothelial permeability compared with administration of MSCs alone [45]. These findings demonstrate a potential role for MSC-based Ang1 therapy in managing patients with ARDS.

## 11. Soluble IL-1 Receptor-Like-1

Interleukin-33 (IL-33), the newest member of the IL-1 family, is produced in response to endothelial and epithelial injury. The axis of IL-33 and its receptor soluble IL-1 receptor-like-1 (sST2) participates in the stimulation and amplification of immunoregulatory and anti-inflammatory responses. Of note, sST2 has been suggested to function as a negative regulator of LPS-induced proinflammatory factor production by acting directly on macrophages through inhibition of the Toll-like receptor 4 expression [46]. Martínez-González et al. engineered adipose-derived MSCs overexpressing sST2 via lentiviral transfection and applied sST2-MSCs in a murine model of LPS-induced ALI [47]. In their study, MSCs overexpressing sST2 afforded a superior therapeutic effect on the detrimental immune-inflammatory response occurring in ALI.

## 12. Interleukin-10

IL-10 is an anti-inflammatory factor with vital immunoregulation effects. IL-10 expression is severely reduced in mice suffering from ALI, and a strategy aimed at increasing the level of IL-10 in the local microenvironment might alleviate lung injury. Kapur et al. demonstrated that an inhibitory response mediated by both T regulatory cells (Tregs) and dendritic cells (DCs) defended against transfusion-related ALI via IL-10. Furthermore, IL-10 administration fully prevented and rescued lung injury in mice [48]. Intriguingly, Wang et al. demonstrated that administration of MSCs overexpressing IL-10 resulted in a sustainably higher IL-10 concentration in serum compared to direct IL-10 injection, thereby inducing a series of positive regulatory effects associated with inflammatory reactions and facilitating the survival of endotoxin-induced ALI in a mouse model [49].

## 13. Manganese Superoxide Dismutase

Manganese superoxide dismutase (MnSOD) is a crucial member of the superoxide dismutase (SOD) family that detoxifies reactive oxygen species (ROS), thereby protecting the cells against the detrimental effect of oxidative stress. It is important to note that administration of MnSOD-plasmid/liposome complexes ahead of irradiation-induced lung injury can alleviate the injury caused by acute or chronic irradiation [50]. In mouse models of radiation-induced lung injury (RILI), systemic administration of bone marrow-derived MSCs overexpressing MnSOD via lentiviral transfection markedly reduced oxidative stress, ameliorated lung inflammation, attenuated pulmonary fibrosis, improved histopathologic changes, and protected the lung cells from apoptosis [51].

## 14. Keratinocyte Growth Factor

Keratinocyte growth factor (KGF), a paracrine cytokine belonging to the fibroblast growth factor (FGF) family, is significantly upregulated following epithelial damage, suggesting that it has a vital role in tissue repair [52]. Increasing evidence has shown that KGF can protect lung tissues from oxidative insults and facilitate the regeneration of type II alveolar epithelial cells by acting on a subset of FGF receptor isoforms, thereby maintaining the integrity of the alveolar barrier [53]. Interestingly, Chen et al. demonstrated that MSC-based KGF gene therapy not only ameliorated pulmonary microvascular permeability but also attenuated proinflammatory responses in a mouse model of ALI induced by LPS [54]. The synergistic effects of KGF-MSCs on lung injury may be ascribed to the stimulation of type II alveolar cell proliferation.

## 15. Developmental Endothelial Locus-1

Developmental endothelial locus-1 (Del-1) is an endothelial-secreted anti-inflammatory molecule that inhibits inflammatory cell recruitment and the integrin lymphocyte function-associated antigen-1-dependent leukocyte-endothelial adhesion [55]. Del-1-overexpressed MSCs were established via lentiviral vectors and administered into mice of ALI injured by LPS through the tail vein; researchers found that the administration with Del-1-overexpressed MSCs offered incremental benefits on LPS-induced ALI [56]. There was a marked reduction in the serum levels of IL-6 and TNF-*α* and the number of neutrophils in BAL in the ALI mice treated with MSCs carrying Del-1. Moreover, lower lung injury scores and a higher myeloperoxidase activity were observed in the mice treated with Del-1-expressed MSCs than in the mice treated with MSCs alone.

## 16. The Limitations of Genetic Modification

Owing to its high transfection efficiency, viral transfection is the most common method used for genetic modification. However, there are several drawbacks to viral transfection. Firstly, viral transfection has the risk of triggering oncogenes during the transgene procedure; thus, it may lead to tumorigenesis [57]. Secondly, viral vectors may evoke antigen-specific adaptive immune responses which can scavenge therapeutic gene products and transfected MSCs [58], ultimately limiting the therapeutic efficacy. Thirdly, it usually takes weeks or months to establish genetically modified MSCs and harvest enough cells for transplantation. However, ARDS is an acute disease which progresses rapidly within days or even within hours. Thus, it may be hard to administrate genetically modified MSCs immediately following the onset of ARDS. Finally, there is still a long way to establish regulatory criteria regarding the procedure of genetically modified MSCs and administration protocols.

## 17. Preconditioning Strategies to Improve MSC Efficacy

Apart from genetic modification, a series of preconditioning strategies has been developed to enhance the therapeutic effect of MSCs in animal ARDS models, including preconditioning with hypoxia, serum from ARDS patients, N-acetylcysteine, TGF-*β*, and 3-dimensional culture. These preconditioning strategies are summarized in Table 3.

## 18. Hypoxia

The oxygen tension niche plays a crucial role in retaining MSC properties and functions. MSCs are routinely cultured under normoxic oxygen tension (21% O_2_) in vitro prior to transplantation, but such a cell cultivation environment cannot mimic the in vivo hypoxic environment. The physiological oxygen tension in the bone marrow environment and other tissues is hypoxic, ranging from 1% to 12% O_2_. MSCs cultured under hypoxic conditions can maintain their multipotent phenotype, increase proliferation, and inhibit senescence [59, 60]. In addition, it is well documented that preexposure to hypoxia prepares MSCs for the hypoxic conditions encountered in ischemic microenvironments following transplantation, thereby reducing hypoxia-induced cellular apoptosis [61].

Paracrine action is one of the best-documented properties of MSCs and exerts a pivotal role in the beneficial effects of MSC administration. The soluble factor profile of MSCs is extremely affected by the local microenvironment surrounding the cells, and some trophic cytokines are considerably upregulated in response to pathological stimulation [62]. Hypoxic culture triggers MSCs to secrete an abundance of soluble factors, including VEGF, Ang1, HGF, IGF-1, and bal-2, which are bioactive molecules associated with proangiogenic, antiapoptotic, and antioxidative effects [63, 64]. In a bleomycin-induced pulmonary fibrotic mouse model, the administration of hypoxia-preconditioned MSCs significantly downregulated the levels of inflammatory and fibrotic factors in the lung tissues and attenuated the degree of lung fibrosis [65]. These observed benefits were partially attributed to the upregulation of the hepatocyte growth factor under hypoxic conditions.

Mounting evidence suggests that pretreatment with hypoxia facilitates migration of MSCs by upregulating the level of SDF-1 as well as stimulating CXCR4 expression [66]. Short-term culture of MSCs under 1% oxygen upregulated the mRNA and protein of CXCR4 [67]. In line with these findings, hypoxic preconditioning evoked MSCs to express high levels of CXCR4 and CXCR7, the receptors of the chemokine stromal-derived factor-1 (SDF-1), by activating hypoxia-inducible factor-1 (HIF-1*α*), thereby improving the adhesion and engraftment of MSCs in the target tissue [68]. Consequently, optimizing the oxygen concentration prior to MSC administration should be deemed a novel strategy to enhance their engraftment in vivo.

Hypoxia preconditioning combined with MSC transplantation to achieve increased therapeutic effects has been extensively tested in a number of preclinical disease models, including brain ischemia, myocardial infarction, and acute kidney injury [69–71]. Treatment with hypoxia-preconditioned MSCs extended the duration of survival of engrafted cells, improved pulmonary respiratory functions, downregulated inflammatory and fibrotic factor expression, and alleviated histological changes in a bleomycin-induced pulmonary fibrosis mouse model [65]. Liu et al. investigated the therapeutic impact of hypoxia-preconditioned MSCs on ischemia/reperfusion (I/R) lung injury in a rat model and found that hypoxic MSCs quickly moved towards injured lung tissues and mitigated pulmonary damage through anti-inflammatory, antiapoptotic, and antioxidant mechanisms [72].

## 19. Serum from ARDS Patients

Recently, the study performed by Islam et al. found that distinct proteomic profiles were observed in different stages of lung injury, and the lung microenvironment is a crucial determinant of effective MSC therapy in ALI [73], highlighting the significance of taking into account MSC-microenvironment interactions when applying MSC therapy in ARDS. MSCs activated with a pool of serum obtained from patients with ARDS led to increased IL-10 and interleukin-1 receptor antagonist (IL-1RN) expression, thus improving the protective anti-inflammatory capacity of MSCs compared with nonactivated cells [74]. In a murine model of ARDS, administration of these ARDS serum-preactivated MSCs was more effective in reducing lung injury score, attenuating pulmonary edema, and alleviating inflammatory cell accumulation compared with control MSCs [74].

## 20. N-Acetylcysteine

N-Acetylcysteine (NAC) is a precursor of glutathione and has antioxidative action against the toxic effects of reactive oxygen species (ROS) by scavenging free radicals and conferring substrates to activate antioxidant enzymes. Wang et al. demonstrated that NAC preconditioning can restore cellular redox balance by eliminating ROS and increasing glutathione levels when MSCs are exposed to oxidative stresses in vitro [75]. Furthermore, NAC pretreatment strengthened the therapeutic effect of the transplanted MSCs in a mouse model of bleomycin-induced lung injury. MSCs pretreated with NAC were more effective than control cells at improving the engraftment and survival rate of MSCs in injured lung tissue while reducing the pathological grade of pulmonary inflammation and fibrosis [75].

## 21. Transforming Growth Factor-*β*1

The chemotaxis and homing of MSCs in vivo are critical to the local microenvironment, particularly the extracellular matrix (ECM), which may enable MSC survival and expansion. Li et al. demonstrated that pretreatment of MSCs with low levels of TGF-*β*1 resulted in increased expression of fibronectin, which is a major component of ECM [76]. To further explore whether pretreatment with a low concentration of transforming growth factor-*β*1 (TGF-*β*1) was favourable for MSC survival in vivo, a rat model of LPS-induced ALI was generated, and the results showed that an increased number of MSCs were observed in the lung 2 weeks following transplantation, indicating that TGF-*β*1-treated MSCs may enhance their long-term therapeutic effect when applied to tissue repair [76].

## 22. Three-Dimensional Culture

Cultivation of MSCs in a three-dimensional (3D) microenvironment is a novel preconditioning strategy to replicate the physiological or pathological milieu where the cells would reside following transplantation [77]. Bartosh et al. suggested that the 3D culture of MSCs in spheroids was more effective than MSCs from adherent cultures in attenuating neutrophil activity and reducing proinflammatory cytokines in a mouse model of peritonitis, indicating that MSC culture in a 3D condition is a promising approach for diseases featuring unresolved inflammation [78]. Other studies found that adipose-derived stem cells exposed to short-term spheroid formation before the monolayer culture exhibited superior regenerative potential by improving their chemotaxis, angiogenesis, and stemness properties, thus enhancing their repair capacity for clinical application [79].

## 23. Other Preconditioning Strategies to Increase MSC Potency

There are also a variety of preconditioning strategies that have been tested in other disease models. Pioglitazone, a peroxisome proliferator-activated receptor-*γ* (PPAR-*γ*), is known to trigger metabolism of mitochondrial free fatty acid, which is a major energy source for cardiomyocytes. Administration of pioglitazone-pretreated MSCs significantly improved efficiency of cardiomyogenesis and cardiac function as evidenced by echocardiogram and immunohistochemistry results [80]. Pretreatment of MSCs with tetrandrine prior to transplantation can significantly enhance the immunomodulation efficacy. In a mouse ear skin inflammation model, systemic administration of tetrandrine-pretreated MSCs markedly attenuated the level of TNF-*α* in the inflamed ear, compared to unpretreated MSCs [81]. These precondition strategies might be promising for increasing MSC potency in the management of ARDS.

## 24. Other Strategies to Optimize MSC Therapy

MSCs gradually lose their initial morphology and multiple-differentiation ability over time. In addition, long-term culture will result in abnormal changes to DNA, RNA, and protein in MSCs [82]. After more than 15 passages, both the proliferation and the bone formation ability of MSCs are significantly reduced. Additionally, the chemokine receptors and paracrine cytokines expressed by aged MSCs are markedly decreased, which in turn attenuates MSCs' migration capacity and pleiotropic properties [83]. Now, it is believed that the proliferation capacity, paracrine signaling, differentiation potential, and DNA stability of MSCs within five serial passages are all maintained in relatively good condition and that such MSCs may achieve better therapeutic effects when applied to patients with ARDS than aged MSCs.

Recently, Islam et al. demonstrated that the lung microenvironment is a major determinant of the beneficial or detrimental effects of MSCs and that the time window of administration is an important factor when initiating MSC therapy in patients with ARDS [73]. In their study, MSCs were shown to aggravate acid-induced lung damage related to pulmonary fibrosis in the lung microenvironment, in which high levels of IL-6 and fibronectin were observed. Notably, modulation of the lung microenvironment with glutathione peroxidase-1 could reverse the deleterious effects of MSCs [73]. However, the lung tissues in different parts had diverse pathological changes due to the heterogeneity of ARDS. Consequently, determining the optimal local microenvironment and the ideal time window for MSC transplantation requires further research.

ARDS is a heterogeneous syndrome resulting from various aetiologies. A latent class analysis with data from two randomized controlled trials confirmed the presence of two ARDS subphenotypes, one of which was characterized by higher plasma levels of inflammatory biomarkers [84]. Response to treatment with positive end-expiratory pressure and fluid management strategies differed on the basis of subphenotype [84, 85]. H1N1 influenza-mediated lung injury in mice was unresponsive to MSC therapy [86]; however, MSCs markedly attenuated the injury of the alveolar-capillary membrane barrier in the more inflammatory H5N1-infected mice and improved their likelihood of survival [87]. In theory, ARDS patients with a hyperinflammatory endotype are more likely to benefit from MSC therapy. In future clinical trials, identification and selection of populations with hyperinflammatory endotypes might be crucial for effective MSC therapy in patients with ARDS.

## 25. Conclusion

MSC-based therapy offers great promise for the management of ARDS due to its anti-inflammatory, antioxidative, immunomodulatory, antiapoptotic, and proangiogenic properties. However, the low mobilization of MSCs to the sites of injury and poor survival of transplanted MSCs in the harsh microenvironment are obstacles faced by clinical translation. In recent years, several strategies have been tested in preclinical models to improve the therapeutic efficacy of MSCs, including genetic manipulation, hypoxia preconditioning, and modulation of the lung microenvironment. Despite increasing evidence that these strategies significantly enhance the innate properties of MSCs, thereby enhancing tissue repair and restoring lung function, further researches are still needed before these strategies can be translated into clinical practices.

## Figures and Tables

**Table 1:** The completed clinical studies regarding the treatment of ARDS with MSCs.

Study title	The number of included patients	MSC source	MSC dose	Outcomes	Significance	Reference
Treatment of acute respiratory distress syndrome with allogeneic adipose-derived mesenchymal stem cells: a randomized, placebo-controlled pilot study	12	MSCs derived from adipose of a healthy female donor	One dose; 1 × 10^6^ cells/kg	(i) No patient suffered clinical complications related to cell infusion (ii) No significant differences regarding PaO_2_/FiO_2_ and SP-D, IL-6, or IL-8 levels were observed between the MSC group and the placebo group	Infusion of allogeneic adipose-derived MSCs was safe in patients with ARDS	[27]

Mesenchymal stem (stromal) cells for treatment of ARDS: a phase 1 clinical trial	9	MSCs derived from the bone marrow of a healthy male donor	One dose; three patients received 1 × 10^6^ cells/kg PBW; three patients received 5 × 10^6^ cells/kg PBW; three patients received 10 × 10^6^ cells/kg PBW	(i) No infusion-related events or treatment-associated adverse events were observed (ii) The LIS and SOFA score were lower in the high-dose group, but did not differ significantly compared with both reduced doses	All three doses of MSCs were safe in patients with moderate-to-severe ARDS	[28]

In vivo effects of mesenchymal stromal cells in two patients with severe acute respiratory distress syndrome	2	MSCs derived from the bone marrow of a healthy male volunteer	One dose; 2 × 10^6^ cells/kg	(i) A reduction in multiple pulmonary and systemic markers of inflammation (ii) Both patients demonstrated improved lung function	MSCs might have clinical efficacy in severe refractory ARDS	[29]

Treatment with allogeneic mesenchymal stromal cells for moderate to severe acute respiratory distress syndrome (START study): a randomized phase 2a safety trial	60	Allogeneic MSCs derived from human bone marrow	One dose; 10 × 10^6^ cells/kg PBW	(i) No infusion-related hemodynamic or respiratory adverse events were observed (ii) Mortality was higher in the MSC group than in the placebo, but did not differ significantly between groups	One dose of intravenous MSCs was safe in patients with moderate to severe ARDS	[30]

LIS: lung injury score; SOFA: Sequential Organ Failure Assessment; PBW: predicted bodyweight; SP-D: serum surfactant-associated protein-D; IL-6: interleukin-6; IL-8: interleukin-8.

**Table 2:** Genetic modification to enhance MSC potency in ARDS preclinical model.

Candidate gene	ARDS preclinical model	MSC source/delivery route	Effects	Reference
CXCR4	Rat model of LPS-induced ALI	Bone marrow/tail vein injection	(i) Facilitate homing of MSCs to damaged lung tissue (ii) Enhance MSC inhibition of lung injury (iii) Enhance the inhibition of lung tissue inflammation	[31]

EP2	Murine model of LPS-induced ALI	Bone marrow/tail vein injection	(i) Improve MSC retention in the lung (ii) Reduce LPS-induced pulmonary vascular permeability (iii) Improve lung histopathology	[33]

HO-1	Rat model of LPS-induced ALI	Bone marrow/tail vein injection	(i) Attenuate LPS-induced lung injury (ii) Increase the levels of HGF, KGF, and IL-10 (iii) Improve 7-day survival rate	[35]

ACE2	Murine model of LPS-induced ALI	Bone marrow/tail vein injection	(i) Improve lung histopathology (ii) Alleviate LPS-induced lung and systemic inflammation (iii) Improve pulmonary endothelial functions	[39]

HGF	Mouse model of radiation-inducedlung injury	Bone marrow/tail vein injection	(i) Attenuate histopathological changes (ii) Improve lung permeability (iii) Reduce secretion and expression of proinflammatory cytokines	[42]

Ang1	Murine model of LPS-induced ALI	Bone marrow/jugular vein injection	(i) Improve lung histopathology (ii) Attenuate the increase in MPO activity (iii) Reduce neutrophil cell count in BALF	[45]

sST2	Murine model of LPS-induced ALI	Adipose tissue/tail vein injection	(i) Attenuated pulmonary inflammation (ii) Decreased apoptosis and necrosis of bronchial tissue	[47]

IL-10	Mouse model of endotoxin-induced ALI	Bone marrow/intratracheal injection	(i) Promote better survival in ALI mice (ii) Reduce BALF protein level (iii) Result in sustained enrichment of serum IL-10 and IL-10-expressing T cells	[49]

MnSOD	Mouse model of radiation-induced ALI	Bone marrow/tail vein injection	(i) Improved survival and lung histopathology injury (ii) Alleviate lung inflammation (iii) Exert antifibrotic effect	[51]

KGF	Mouse model of LPS-induced ALI	Bone marrow/tail vein injection	(i) Reduce lung wet/dry ratio (ii) Improve lung inflammation (iii) Improve lung histopathology and survival	[54]

Del-1	Mouse model of LPS-induced ALI	Bone marrow/tail vein injection	(i) Reduce lung wet/dry ratio (ii) Attenuate the increase in MPO activity (iii) Alleviate lung inflammation	[56]

ARDS: acute respiratory distress syndrome; ALI: acute lung injury; LPS: lipopolysaccharide; CXCR4: chemokine receptor 4; EP2: E-prostanoid 2 receptor; HO-1: heme oxygenase-1; ACE2: angiotensin-converting enzyme 2; HGF: hepatocyte growth factor; Ang1: angiopoietin-1; sST2: soluble IL-1 receptor-like-1; IL-10: interleukin-10; MnSOD: manganese superoxide dismutase; KGF: keratinocyte growth factor; BALF: bronchoalveolar lavage fluid; MPO: myeloperoxidase; Del-1: developmental endothelial locus-1.

**Table 3:** Different preconditioning strategies to improve MSC efficacy in the ARDS preclinical model.

Preconditioning	ARDS preclinical model	MSC source/delivery route	Effects	Reference
Hypoxia	Rat model of ischemia/reperfusion-induced lung injury	Rat bone marrow/administration in the circulating perfusate into pulmonary artery	(i) Reduce lung weight gain and the ratio of wet weight/dry weight (ii) Decrease white cell count in BALF (iii) Reduce level of MPO in the lung tissue	[72]

Serum from ARDS patients	Murine model of LPS-induced ARDS	Human bone marrow/intravenous injection	(i) Reduce BALF inflammatory cells (ii) Increase plasma IL-10 (iii) Decrease TNF and IL-1	[74]

NAC	Mice model of bleomycin-induced lung injury	Human embryo/tail vein injection	(i) Reduce inflammation and fibrosis in the injured lung (ii) Reduction of apoptosis in lung cells (iii) Reducing the mortality of mice with bleomycin-induced lung injury	[75]

TGF-*β*1	Rat model of LPS-induced ALI	Human umbilical cord/tail vein injection	(i) Attenuate LPS-induced systemic injury (ii) Increase MSC survival in damaged lungs	[76]

ARDS: acute respiratory distress syndrome; ALI: acute lung injury; LPS: lipopolysaccharide; NAC: N-acetylcysteine; TGF-*β*1: transforming growth factor-*β*1; BALF: bronchoalveolar lavage fluid; MPO: myeloperoxidase; IL-10: interleukin-10; IL-1: interleukin-1.
